# LINE Insertion Polymorphisms are Abundant but at Low Frequencies across Populations of *Anolis carolinensis*

**DOI:** 10.3389/fgene.2017.00044

**Published:** 2017-04-13

**Authors:** Robert P. Ruggiero, Yann Bourgeois, Stéphane Boissinot

**Affiliations:** New York University Abu DhabiAbu Dhabi, United Arab Emirates

**Keywords:** retrotransposon, LINE, *Anolis carolinensis*, genome resequencing, transposable element, selection

## Abstract

Vertebrate genomes differ considerably in size and structure. Among the features that show the most variation is the abundance of Long Interspersed Nuclear Elements (LINEs). Mammalian genomes contain 100,000s LINEs that belong to a single clade, L1, and in most species a single family is usually active at a time. In contrast, non-mammalian vertebrates (fish, amphibians and reptiles) contain multiple active families, belonging to several clades, but each of them is represented by a small number of recently inserted copies. It is unclear why vertebrate genomes harbor such drastic differences in LINE composition. To address this issue, we conducted whole genome resequencing to investigate the population genomics of LINEs across 13 genomes of the lizard *Anolis carolinensis* sampled from two geographically and genetically distinct populations in the Eastern Florida and the Gulf Atlantic regions of the United States. We used the Mobile Element Locator Tool to identify and genotype polymorphic insertions from five major clades of LINEs (CR1, L1, L2, RTE and R4) and the 41 subfamilies that constitute them. Across these groups we found large variation in the frequency of polymorphic insertions and the observed length distributions of these insertions, suggesting these groups vary in their activity and how frequently they successfully generate full-length, potentially active copies. Though we found an abundance of polymorphic insertions (over 45,000) most of these were observed at low frequencies and typically appeared as singletons. Site frequency spectra for most LINEs showed a significant shift toward low frequency alleles compared to the spectra observed for total genomic single nucleotide polymorphisms. Using Tajima’s D, *F*_ST_ and the mean number of pairwise differences in LINE insertion polymorphisms, we found evidence that negative selection is acting on LINE families in a length-dependent manner, its effects being stronger in the larger Eastern Florida population. Our results suggest that a large effective population size and negative selection limit the expansion of polymorphic LINE insertions across these populations and that the probability of LINE polymorphisms reaching fixation is extremely low.

## Introduction

The complete sequencing of dozens of vertebrate genomes representing most extant lineages has been an extraordinary source of information, thereby revolutionizing the field of genetics, development and evolutionary biology. However, those genomes vary considerably in size and structure and understanding the cause(s) of these differences is fundamental for meaningfully interpreting genomic annotations (Elliott and Gregory, 2015). Among the features that show the most variation across vertebrate taxa is the abundance and diversity of non-LTR retrotransposons [also called LINEs for Long Interspersed Nuclear Elements; reviewed in Tollis and Boissinot (2012)]. LINEs are autonomously replicating retroelements, meaning they encode the molecular machinery necessary for their own replication. LINEs are ubiquitous components of eukaryotic genomes and the origin of the main LINE lineages is very ancient, possibly predating the origin of eukaryotes (Malik et al., 1999). LINEs are classified into a number of clades based on the presence of conserved features (Malik et al., 1999; Kapitonov et al., 2009). The most basal clades of LINEs (e.g., R2, R4, RTE) contain a single open-reading frame (ORF) encoding a reverse transcriptase domain, while the most derived lineages contains two ORFs (e.g., L1, L2, CR1). The mechanism of transposition was characterized for the R2 and L1 elements and it is assumed that other LINEs transpose using a similar mechanism (Luan et al., 1993; Cost et al., 2002). Following transcription and export of LINE mRNA to the cytoplasm, LINE-encoded proteins are translated and form an RNA-protein complex that is reimported in the nucleus. In the nucleus, reverse transcription takes place at the site of insertion, through a process called target-primed reverse transcription. Although there is a strong *cis* preference (Wei et al., 2001), the replicative machinery of LINEs can act on other transcripts and is responsible for the amplification of the non-autonomous SINEs and of retrotransposed pseudogenes (Ohshima et al., 1996; Dewannieux et al., 2003; Dewannieux and Heidmann, 2005; Piskurek et al., 2009).

Long Interspersed Nuclear Elements are ubiquitous in vertebrates and constitute the dominant category of autonomously replicating retroelements in most vertebrate genomes (Tollis and Boissinot, 2012). They have considerably affected the size and structure of these genomes and it is believed that LINE abundance is one of the major determinants of haploid genome size differences among vertebrates. At one extreme, mammalian genomes contain extremely large numbers of LINEs that can account for as much as 30% of their size (Lander et al., 2001; Waterston et al., 2002). LINEs in placental mammals are represented by a single clade, L1. The vast majority of L1 elements are the product of past amplification and in most species only the most recently evolved family of elements is active at a time (Furano, 2000). Fish, amphibians and non-avian reptile genomes contain a much larger diversity of active LINE families, generally representing multiple clades (Volff et al., 2003; Duvernell et al., 2004; Furano et al., 2004; Novick et al., 2009; Blass et al., 2012; Chalopin et al., 2015). These families are usually represented by small numbers of very similar copies, suggesting that the majority of insertions are recent (Furano et al., 2004; Novick et al., 2009; Blass et al., 2012).

In mammals, the evolutionary dynamics of LINEs is relatively well understood. Population genetics and genomics studies in humans have shown that the majority of L1 elements behave as neutral alleles and accumulate readily in the genome of their host (Boissinot et al., 2006). This does not mean that L1 activity is fully neutral. In humans, a fitness cost related to the length of L1 elements has been demonstrated (Boissinot et al., 2001, 2006). This suggests that the deleterious effect of L1 result from the ability of long elements to mediate ectopic recombination events (Myers et al., 2005; Song and Boissinot, 2007). However, this cost is insufficient to prevent the fixation of most elements, hence the extremely large number of L1 copies in mammals. By comparison the dynamics of LINEs in non-mammalian genomes is not as well understood. The young age and relatively small number of LINEs in fish and reptile genomes could be interpreted as evidence for a lower rate of fixation of novel insertions in non-mammalian genomes. Studies in stickleback and in lizard suggest that, indeed, LINE insertions tend to be negatively selected, yet a number of insertions do reach fixation (Blass et al., 2012; Tollis and Boissinot, 2013). In addition, population genetics data in the pufferfish show that the frequency spectrum of recent insertions is consistent with neutrality (Neafsey et al., 2004). Thus we have been unable to exclude the possibility that LINEs are neutral or weakly deleterious in non-mammalian vertebrates and that their copy number is controlled by other means, possibly by a faster decay due to a higher rate of DNA loss (Novick et al., 2009; Blass et al., 2012).

At this point, our understanding of LINE population dynamics is heavily biased toward their dynamics in humans. However, the extreme abundance and low diversity of LINEs in mammals constitute a derived state relative to other vertebrates. Thus, inferences drawn from studies in mammals are unlikely to apply to other vertebrates. In addition, results obtained from previous studies in non-mammalian vertebrates provide only a partial picture since those studies relied on a relatively small number of polymorphisms, principally collected from the published reference genomes (Neafsey et al., 2004; Blass et al., 2012; Shen et al., 2013; Tollis and Boissinot, 2013). Thus, we decided to investigate the population dynamics of LINEs in a non-mammalian vertebrate, the green anole *Anolis carolinensis*, using a complete genome re-sequencing approach. The anole genome is a particularly good model because it is among the most diverse vertebrate genomes in terms of LINE diversity (Novick et al., 2009; Chalopin et al., 2015). Five LINE clades are simultaneously active in anole: L1, L2, CR1, R4 and RTE. These elements differ considerably in structure, copy number, and diversity (**Table 1**). For example, the L1 and the L2 clades contain 20 and 17 highly divergent families, respectively, whereas the CR1 clade is represented by only 4 closely related families. Since these clades and families coexist within the same genome, they are equally affected by the demography of their host. It is thus possible to assess their relative impact on fitness and to infer the evolutionary processes determining their diversification and replicative success.

**Table 1:** Long Interspersed Nuclear Element clades found in the *A. carolinensis* genome.

Clades	Number of families	Number of RT hits^1^	Total number of copies in published genome^1^	Number of full-length copies in published genome^1^	Length of full length elements^1^	Number of polymorphic insertions^2^	Number of full-length polymorphic insertions^2^
R4	2	7,682	3,000	994	3.8 Kb	1,729	712
RTE	2	18,554	3,516	217	3.2–3.9 Kb	3,367	1782
CR1	4	86,802	1,594	117	4.6–5.8 Kb	27,802	2,578
L2	17	38,607	3,800	380	4.8–6.3 Kb	11,210	769
L1	20	7,441	806	170	5.2–6.8 Kb	2,508	1,089


*^1^Data from Novick et al. (2009); ^2^This study.*

In this article we present the first population genetic analysis of LINEs using re-sequencing data in a non-mammalian vertebrate. We sequenced thirteen individuals, from two populations with different demographic histories, at a depth of coverage ranging from 8 to 16×. For each resequenced genome we then characterized the single nucleotide polymorphisms (SNPs) and polymorphic sites containing LINE insertions not found in the reference genome. We determined that the number of insertion polymorphisms generated by LINEs in this species is large, exceeding 45,000 insertions, with substantial differences in replicative success among clades. We also determined that the vast majority of these insertions exist at very low frequency in natural populations as a result of the very large effective population size of *A. carolinensis* and of purifying selection against those insertions.

## Materials and Methods

### Sampling

There are five geographically and genetically distinct anole populations in North America (Campbell-Staton et al., 2012; Tollis et al., 2012; Tollis and Boissinot, 2014; Manthey et al., 2016). We decided to focus our re-sequencing effort on two of those populations, the Eastern Florida population and the Gulf-Atlantic population (**Table 2**). The Eastern Florida population is restricted to a ∼50 Km band along the eastern coast, extending from Jacksonville in the north to West Palm Beach in the south. Demographically, this population has remained relatively stable during the Pleistocene, with a slight signature of expansion (Manthey et al., 2016). The Gulf-Atlantic population is about 10 times smaller, although it is widely distributed from the Atlantic coast of Georgia and North Carolina to Texas in the west. It has experienced a bottleneck followed by demographic expansion (Manthey et al., 2016). This study was carried out in accordance with the recommendations of the American Veterinary Medical Association for the euthanasia of ectotherms. The protocol was approved by the Queens College Institutional Animal Care and Use Committee (Animal welfare assurance number: A32721-01; protocol number: 135).

**Table 2:** Origin of the samples sequenced, sequencing depth, and number of polymorphic insertions per individual.

Sample	Clade	Locality	Latitude	Longitude	Depth	Number of polymorphic insertions present	Number of polymorphic full-length insertions present
AC_36_1	Gulf-Atlantic	Blount, Tennessee	35.53855	-84.07625	15×	7,557	839
AC_38_4	Gulf-Atlantic	Blount, Tennessee	35.5558	-84.00245	10×	6,367	699
AC_8_13	Gulf-Atlantic	Thibodaux, Louisiana	29.797883	-90.8129	9×	6,402	629
AC_8_8	Gulf-Atlantic	Thibodaux, Louisiana	29.797883	-90.8129	16×	7,849	861
AC_27_3	Gulf-Atlantic	Darien, Georgia	31.35295	-81.447467	10×	5,626	565
AC_27_4	Gulf-Atlantic	Darien, Georgia	31.35295	-81.447467	10×	5,135	500
CC3	East Florida	Cocoa, Florida	28.243611	-80.870556	16×	9,969	863
CC8	East Florida	Cocoa, Florida	28.243611	-80.870556	16×	11,965	1,130
SB3	East Florida	South Bay, Florida	26.683333	-80.716884	12×	11,839	1,069
SB4	East Florida	South Bay, Florida	26.683333	-80.716884	8×	8,371	621
TV8	East Florida	Titusville, Florida	28.5437777	-80.9421666	8×	8,557	740
VB6	East Florida	Vero Beach, Florida	27.640278	-80.59475	10×	10,393	890
VB7	East Florida	Vero Beach, Florida	27.640278	-80.59475	9×	10,451	924


### DNA Extraction and Whole Genome Sequencing

DNA samples were retrieved from ethanol-preserved tissue and isolated with Ampure beads using the manufacturer’s protocol. For each sample 200 ng of DNA was used to prepare Illumina TRU-Seq paired end libraries and sequenced on an Illumina HiSeq 2500, at the NYUAD Center for Genomics And Systems Biology Sequencing Core^1^. Sequencing was conducted twice, once to generate higher depth of coverage (two individuals per lane) and once to generate a broader sampling (four individuals per lane) at lower depth of coverage. Quality assessment was conducted using FastQCv0.11.5^2^ followed by quality trimming. We used Trimmomatic (Bolger et al., 2014) to trim off low quality bases, sequencing adapter contamination and systematic base calling errors. The specific parameters we used were “trimmomatic_adapter.fa:2:30:10 TRAILING:3 LEADING:3 SLIDINGWINDOW:4:15 MINLEN:36.” For the higher depth of coverage runs an average of 1,519,339,234 read pairs were generated: after quality trimming read pairs, we retained 93.3% as paired reads and 6.3% as single reads. For the lower depth of coverage runs an average of 99,464,570 read pairs were generated: after quality trimming read pairs, we retained 89.8% as paired reads and 9.9% as single reads (Supplementary Table S1). Sequencing data from this study have been submitted to the Sequencing Read Archive^3^ under the BioProject designation PRJNA376071.

### Sequence Alignment and SNP Calling

Surviving reads were aligned to the May 2010 assembly of the *A. carolinensis* reference genome (Broad AnoCar2.0/anoCar2; GCA_000090745.1; Alfoldi et al., 2011) and processed for SNP detection with the assistance of the NYUAD Bioinformatics Core, using NYUAD variant calling pipeline. For each sample, quality-trimmed reads were aligned to the reference genome using Bowtie2 (Langmead and Salzberg, 2012). The resulting SAM file for each individual was sorted, converted into BAM format and indexed using SAMtools (Li et al., 2009). These files were then checked for insertions, deletions and duplications using Picard tools^4^ and GATK was applied for indel realignment, SNP and indel discovery and genotyping according to GATK Best Practices (DePristo et al., 2011; Van der Auwera et al., 2013). To maximize the sensitivity and confidence of variant calls, joint genotyping was conducted using GATK. To do this we first generated genomic VCF (g.VCF) files for each individual, then applied the GenotypeGVCFs command, using the previously generated g.VCF as input, to generate a group VCF file containing SNPs for the 13 genomes from the two *Anolis* populations considered here. To confirm the efficacy of this approach we selectively compared high quality genotype calls from the GATK to results from SAMtools *mpileup* (Li et al., 2009).

### SNP Filtering

Our goal was to compare the frequency of polymorphic LINE insertions to the frequency of SNPs across the genome (excluding LINEs), requiring a high confidence collection of SNPs. SNPs were filtered using VCFTOOLS (Danecek et al., 2011), by applying the following criteria: a minimum Phred-score of 20, a minimum sequencing depth of 6× for each genotype, a minimum genotype quality of 20. Indels were removed and only SNPs genotyped in all individuals after quality trimming were kept for further analysis. SNPs were sampled every 1,000 SNPs to limit the effect of linkage disequilibrium while retaining enough markers for precise parameters estimation (332,839 SNPs). Options in VCFTOOLS were thus as follows: –minDP 6 –minGQ 20 –minQ 20 –max-missing 1 –min-alleles 2 –max-alleles 2 –remove-indels. Filtering might lead to biases when estimating the neutral allele frequency spectrum (Kim et al., 2011). However, our filtering criteria did not result in any strong bias in summary statistics when compared to the unfiltered VCF file, suggesting that bias in allele frequency estimates due to filtering remained limited.

### Mobile Element Polymorphism Detection

To characterize LINE insertion polymorphisms, we used the Mobile Element Locator Tool (MELT^5^; Sudmant et al., 2015). MELT identifies, characterizes and genotypes polymorphic transposable element insertions and has been used successfully for extensive analyses of LINE and SINE polymorphisms in the human genome (1000 Genomes Project Consortium et al., 2015; Sudmant et al., 2015). MELT exhibits high precision and recall of LINE insertions in low depth of coverage genomes (Rishishwar et al., 2016). MELT identifies the presence and absence of insertions based on the appearance of target mobile element sequence in split or discordant reads. For our analyses we selected target sequences from previously described, potentially active LINE families from the CR1, L1, L2, R4 and RTE clades (Novick et al., 2009). These sequences were identified based on the presence of a characteristic reverse transcriptase domain using Genome Parsing Suite software (McClure et al., 2005), exist as full length copies in the *Anolis* reference genome and exhibit low divergence (typically less than 2% divergence between copies and consensus sequence), indicative of recent activity by members of these groups (Novick et al., 2009). Previously published consensus sequences for these elements were collected from Repbase (Bao et al., 2015) to be used as target sequences, and cleared of ambiguities, when they occurred, by direct comparison to full-length genomic copies. Based on the low divergence exhibited by these groups (Novick et al., 2009), and our intention to generate a conservative estimate, we selected an acceptable error rate of 2%.

Mobile Element Locator Tool operates on BWA-aligned re-sequenced genomes, so for each *Anolis* sample, quality-trimmed FastQ reads were aligned to the AnoCar2.0 genome using the BWA-mem short read alignment approach (Li and Durbin, 2009). Each BWA-aligned sample genome was sorted and converted to BAM format using Samtools (Li et al., 2009). The MELT Preprocess software was then run on each sample genome BAM file to prepare it for analysis. For our analyses we used the MELT-SPLIT pathway, which consists of four runtime stages: individual analysis (IndivAnalysis), group analyses (GroupAnalysis), genotyping (Genotype) and VCF file construction (makeVCF). Individual analyses identify evidence of target element insertions in BAM files. Results from individual analyses are merged during group analysis, and the pooled data is used to produce improved calls regarding each insertion, including breakpoints, insertion length, strand, and target site duplication. Genotyping is conducted on each genome individually to determine its genotype for each polymorphic locus. Finally, the data from individual genotyping are merged to form a VCF file for the population. For each of the 41 specific LINE subgroup consensus sequences, every BWA-aligned and preprocessed genome was analyzed and used to produce VCF files for individuals from the East Florida and Gulf Atlantic *Anolis* populations. These files were then combined and filtered to remove any polymorphic loci that failed to exhibit coverage in all samples or exhibited low quality calls. Where duplicate calls occurred (i.e., when multiple LINE insertions of different families occurred within 50 bp from each other) only the longest was kept in the VCF file. This study focused exclusively on the presence and predicted length of polymorphic LINE insertions and at no point do we analyze or discuss mutations occurring within these insertions since it is nearly impossible to match a SNP within a LINE with its specific genomic location.

### Descriptive Statistics

We used several statistics to describe the allele frequency spectra and allele sharing between populations, of both SNPs and LINE insertion polymorphisms. Tajima’s D (Tajima, 1989) is a statistic that is commonly used to detect selection. It reflects the difference between θw and π, which are two different estimators of the effective population size scaled by mutation rate (4Neμ) that should be positively correlated under neutrality. At mutation-drift equilibrium, the expected value of Tajima’s D is zero, while positive values indicate population reduction or balancing selection, and negative values indicate population expansion or purifying and positive selection. We computed the mean number of pairwise differences for the whole dataset and each population, as well as the number of private and fixed polymorphisms. We also computed the mean *F*_ST_ between populations for each category of markers. These statistics were calculated using VCFTOOLS (Danecek et al., 2011) and the R package PopGenome (Pfeifer et al., 2014). An element was considered as complete if its size was at least 90% of the maximum size for its family. The vcflib script vcffilter^6^ was used to split VCFs between complete and truncated elements for each family.

### Demographic Parameters Estimation from SNPs

To assess whether LINE variation deviated from a neutral model, we estimated the demographic history of the two populations using the SNP dataset. We fitted a model of isolation with migration, allowing for one population size change in each derived population. Time since divergence between the two species was fixed at 1.34 Mya (Tollis et al., 2012). Parameters were estimated from the joint allele frequency spectrum (SFS) using the likelihood approach implemented in fastsimcoal2.5 (Excoffier et al., 2013). Parameters with the highest likelihood were obtained after 40 cycles of the algorithm, starting with 50,000 coalescent simulations per cycle, and ending with 250,000 simulations. This procedure was replicated 100 times and the set of parameters with the highest final likelihood was retained.

We estimated 95% confidence intervals (CI) by simulating coalescence under the best model for the same number of SNPs as in the original dataset. We performed parameter estimation for 150 of these pseudo-observed datasets to infer CI. Coalescence simulations were performed using fastsimcoal2.5 (Excoffier and Foll, 2011). We further checked whether our model fit the observed data by sampling parameters from the 95% CI range for 10,000 simulations and comparing observed and simulated datasets. We summarized allele frequency spectra using Principal Components Analysis [gfitpca function in the R package abc (Csillery et al., 2012)].

### Simulations and Deviation from Neutral Expectations

To estimate if the LINE SFS deviated significantly from neutral expectations, we simulated for each family the derived allele frequency spectrum in fastsimcoal2.5. Parameters were sampled from the CI obtained for SNPs. We performed 5,000 simulations for each dataset, assuming unlinked LINE insertion sites, and obtained *p*-values from the comparison between the observed Tajima’s D or *F*_ST_ value to the distribution obtained under a neutral model. We also performed a non-parametric bootstrap on the actual SNP dataset and extracted random sets of 100–500 SNPs along each chromosome, computing Tajima’s D and comparing the resulting distribution to the values observed for LINEs.

## Results

### LINE Insertion Polymorphisms are Numerous and Their Abundance Varies by Clade

We sequenced six *A. carolinensis* genomes from the Gulf-Atlantic population and seven from the East Florida population with a sequencing depth of coverage ranging from 8 to 16× after alignment to the reference genome (**Table 2**). We detected extensive LINE insertion polymorphism in both populations (summarized in **Tables 3**, **4**) with a total of 46,616 polymorphic insertions across the 13 individuals. The East Florida population appears to maintain a greater total number of LINE polymorphisms, with a mean of 10,022 polymorphic LINE insertions per individual (from 8,371 to 11,965 insertions). In the Gulf Atlantic population the mean number of insertions per individual was substantially lower at 6,489 (from 5,135 to 7,849 insertions). Across all genomes roughly 10% of all polymorphic insertions approximated their full length, though individual populations varied: for individuals from the Gulf-Atlantic, 10.5% of polymorphic LINE insertions were full length (4,093 out of 38,936), while in the East Florida population only 8.7% (6,237 out of 71,545) were full length.

**Table 3:** Summary statistics for all LINE clades, families and subgroups considered in this study.

		Mean number of differences in polymorphic insertions			Tajima’s D		Number of polymorphic loci	% of private insertions		% of fixed differences	% of shared differences	Mean *F*_ST_
								
Dataset		All	Florida	Gulf-Atl	Florida	Gulf-Atl		Florida	Gulf-Atl
SNPs		0.21	0.22	0.36	-0.62	0.47	314575	60.25	15.85	0.19	23.72	0.12
L1	All	0.15	0.22	0.31	-1.39^∗∗∗^	-0.48^∗∗^	2508	65.67	19.46	0	14.87	0.04^∗∗^
L1_AC1 to 16	FL	0.15	0.21	0.32	-1.46^∗∗∗^	-0.13	454	71.81	18.5	0	9.69	0.04^∗^
	TR	0.18	0.25	0.30	-0.95	-0.5^∗∗^	1062	59.13	15.35	0	25.52	0.04^∗∗∗^
L1_AC17 to 20	FL	0.11	0.17	0.28	-2.06^∗∗∗^	-0.78^∗∗∗^	635	68.82	27.09	0	4.09	0.03^∗^
	TR	0.14	0.20	0.31	-1.6^∗∗∗^	-0.24^∗^	357	71.71	19.33	0	8.96	0.04
L2	All	0.15	0.23	0.28	-1.27^∗∗∗^	-0.74^∗∗∗^	11210	61.06	23.76	0	15.18	0.05^∗∗^
	FL	0.13	0.20	0.28	-1.65^∗∗∗^	-0.75^∗∗∗^	769	67.1	25.1	0	7.80	0.04^∗∗∗^
	TR	0.15	0.23	0.28	-1.24^∗∗∗^	-0.74^∗∗∗^	10440	60.61	23.66	0	15.73	0.05^∗∗^
CR1	All	0.15	0.22	0.31	-1.31^∗∗∗^	-0.29^∗^	27802	70.35	18.02	0.02	11.62	0.05
	FL	0.14	0.21	0.30	-1.51^∗∗∗^	-0.49^∗∗^	2578	68	23.27	0	8.73	0.05
	TR	0.16	0.22	0.31	-1.29^∗∗∗^	-0.27^∗^	25224	70.59	17.48	0.02	11.91	0.05
R4	All	0.17	0.24	0.25	-1.04^∗^	-1.1^∗∗∗^	1729	49.1	20.76	0	30.13	0.03^∗∗∗^
	FL	0.16	0.23	0.25	-1.16^∗∗^	-1.21^∗∗∗^	1017	47.79	20.94	0	31.27	0.02^∗∗∗^
	TR	0.18	0.25	0.27	-0.87	-0.93^∗∗∗^	712	50.98	20.51	0	28.51	0.04^∗∗∗^
RTE-1	All	0.11	0.18	0.23	-1.91^∗∗∗^	-1.42^∗∗∗^	2853	62.57	33.16	0	4.28	0.02^∗∗∗^
	FL	0.11	0.18	0.22	-2.00^∗∗∗^	-1.52^∗∗∗^	1774	61.72	35.17	0	3.10	0.02^∗∗∗^
	TR	0.12	0.19	0.24	-1.77^∗∗∗^	-1.23^∗∗∗^	1079	63.95	29.84	0	6.21	0.02^∗∗∗^
RTEBovB	All	0.25	0.31	0.34	-0.08^+^	0.06	514	37.74	12.84	0	49.42	0.05^∗∗∗^
	FL	0.27	0.38	0.33	0.76	-0.06	8	25	25	0	50.00	0.14
	TR	0.25	0.31	0.34	-0.1	0.06	506	37.94	12.65	0	49.41	0.05


*Mean pairwise divergence was computed only for loci polymorphic within a population and represents the average number of differences in polymorphic insertions or SNPs computed for all pairs of individuals (equivalent to nucleotide diversity). The number of private, shared, and fixed polymorphisms are provided as proportions of sites polymorphic in the whole sample. For each group for which coalescence simulations were performed, we provide a one-tailed *p*-value based on where the observed value for the statistics fell in the simulated distribution. We did not perform separate simulations for truncated and complete elements in RTEBovB due to the low number of polymorphic complete insertions. All, All elements; FL, full-length; TR, truncated; +, Tajima’s D fell in the 0.1% upper tail of the distribution. ^∗^*p*-value < 0.05; ^∗∗^*p*-value < 0.01; ^∗∗∗^*p*-value < 0.001.*

**Table 4:** Copy numbers of L1 and L2 families.

L1 Clade		L2 clade		RTE clade	
		
Families	Copy number	Families	Copy number	Families	Copy number
L1AC01	68	L2AC01	507	RTE-1	2853
L1AC02	18	L2AC02	336	RTEBovB	514
L1AC03	0	L2AC03	301		
L1AC04	43	L2AC04	504		
L1AC05	27	L2AC05	276		
L1AC06	87	L2AC06	569		
L1AC07	532	L2AC07	543		
L1AC08	95	L2AC08	1424		
L1AC09	82	L2AC09	1661		
L1AC10	0	L2AC10	131		
L1AC11	90	L2AC11	720		
L1AC12	52	L2AC12	206		
L1AC13	103	L2AC13	948		
L1AC14	85	L2AC14	256		
L1AC15	181	L2AC15	1177		
L1AC16	53	L2AC16	388		
L1AC17	763	L2AC17	1263		
L1AC18	0				
L1AC19	23				
L1AC20	206				


The five clades of LINEs investigated (R4, RTE, CR1, L1, and L2) exhibited notable variation in their success at generating new insertions (**Tables 1**, **3**). The most successful group was the CR1 clade, for which we found 27,802 polymorphic insertions. The L2 clade also has a large number of insertions: 11,210. Far fewer polymorphisms were found for the remaining families: the RTE clade had 3,367 polymorphisms, the L1 clade 2,508, and the R4 clade 1,729. Within each clade we also found substantial differences in the success of active families (**Table 4**). The L1 clade consists of 20 highly divergent families (Novick et al., 2009; Boissinot and Sookdeo, 2016). We used the consensus sequence for each of these families to search for polymorphisms and found a highly uneven fraction of polymorphic insertions across these families. No polymorphisms were found for three L1 families (L1AC03, L1AC10, and L1AC18), indicating these families are inactive in the populations we studied (**Table 4**). Most families had polymorphic insertions numbering less than 100, however, two families appeared at much higher numbers: L1AC07, which had 532 polymorphic insertions, and L1AC17, which had 763 polymorphic insertions. Together, these two families account for the majority (52%) of all L1 polymorphic insertions we identified. The L2 Clade has 17 known families in the *Anolis* genome but their differences in replicative success were not as large as those in the L1 clade. All L2 families exhibit polymorphisms and the most frequent group, L2AC09, only constitute 14% of L2 insertions. The RTE clade has only two representatives, RTE-1 and the ancient RTEBovB family. There are nearly six times more RTE-1 polymorphisms than RTEBovB (2853 versus 514, respectively), which is consistent with the idea that RTEBovB may be extinct in *Anolis*. The two R4 and the four CR1 families previously described (Novick et al., 2009) are nearly identical in sequence over most of their length and it was not possible to distinguish them using this dataset.

Our prior expectations for the complement of LINE insertions have been shaped in part by published analyses conducted on the *Anolis* genome assembly using GPS-RT (McClure et al., 2005) and by BLAST searches using the 3′ termini of consensus sequences (Novick et al., 2009). Those two earlier analyses were conducted on a single sequence assembly, representing an individual. We compared our results to the results of these earlier analyses to assess how much LINE-generated polymorphisms there are in natural populations relative to the reference genome. The number of polymorphic CR1 insertions we identified is more than 17-fold the total number of insertions from the BLAST search of the reference genome (**Table 1**). This discrepancy is best explained by the large number of severely truncated insertions (<50 bp) that could have been missed by the BLAST search (which used the entire 3′UTR). The number of polymorphic insertions from the L2 clade is slightly less than threefold the number of insertions in the published genome. This is similar to L1, which has just over threefold more polymorphic insertions than insertions in the reference genome, though L1 has far fewer total insertions than L2 (2,500 L1 versus 11,000, respectively). Roughly the same number (∼3,300) of polymorphic RTE insertions were found as were previously detected by BLAST, and the R4 clade was found to have less than half as many polymorphic insertions as insertions identified by BLAST. These differences among clades possibly reflect differences in the fractions of fixed insertions relative to polymorphic ones among clades, which could be due to differential chance of fixation or to different timing of amplification of the LINE clades during the evolution of *A. carolinensis*. The number of RT hits detected by GPS are 3–5 times higher than the number of polymorphisms but the numbers are roughly proportional in the sense that the clades with the largest number of polymorphisms (CR1 and L2) are also the clades with the most RT hits. This difference in the total number of counts probably reflects the ability of GPS to identify the entire complement of RT-containing elements, including ancient elements that have long been fixed in the *Anolis* genome.

### LINE Clades Show Distinct Patterns of Insertion Length and Success

**FIGURE 1:**
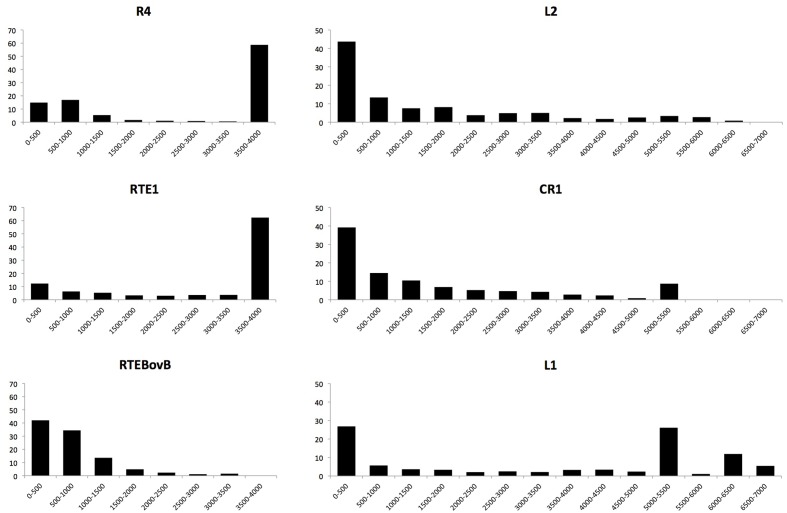
**Length distribution of polymorphic LINE insertions.** The majority of R4 and RT1 insertions approach full-length, while all other LINEs were primarily found as truncated copies. Note that the L1 clade has multiple subfamilies whose full-length copies vary in length and this results in the multiple peaks at the right of the length distribution.

The total number of polymorphic insertions found for each clade is not directly related to the number of full-length insertions. In most clades (with the notable exception of RTE) more truncated than full-length elements were found. All the truncated elements had their 3′ extremity and were truncated in 5′. This pattern is typical of LINEs and is caused by premature termination of the reverse-transcription reaction at the site of insertion (Ostertag and Kazazian, 2001; Martin et al., 2005). The CR1 clade has the largest number of insertions but the fraction of full-length CR1 insertions is less than 10%. For the L2 clade, which is also abundant, less than 7% of these insertions were full-length (769). In contrast, the majority (53%) of the RTE insertions are full-length and ∼40% of the L1 and R4 insertions are complete. It is unlikely that the differences we observe result from differences in the length of LINEs. L1 consensus sequences are the longest (5.2–6.8 kb) whereas the R4 consensus is substantially shorter (3.8 kb), yet the same fraction of insertions is full-length in these two clades. The consensus sequences of L1 and L2 are of similar length but the fraction of full-length insertions is six times larger for L1 than for L2. These differences are likely due to variations in the mode of truncation of the elements at the time of insertions. **Figure 1** depicts the length distribution of the different clades. It shows that truncation in R4 and RTE1 can occur anywhere along the length of the element but a large fraction of the elements are transposed all the way to their 5′ end. The probability of truncation in CR1 and L2 decreases proportionally to the distance to the 3′ end and a minority of the elements insert as full-length. L1 elements either truncate early on during transposition (and don’t reach 1 Kb), or if they do, they tend to be complete, hence the large fraction of elements longer than 5 Kb. It should be noted that complete elements fall into two length categories: elements between 5 and 5.5 Kb and elements longer than 6 Kb. These two types correspond to two sub-clades of L1, the families with short (∼230 bp long) 5′UTR (families L1AC16 to 20) and the families with long (800–1,500 bp) 5′UTRs (families L1AC1 to 15) (Boissinot and Sookdeo, 2016). Finally, the RTEBovB family contains a very small number of full-length elements, which is probably related to the fact that this family is on its way to extinction.

### Most Polymorphic LINE Insertions Exist at Low Frequencies

**FIGURE 2:**
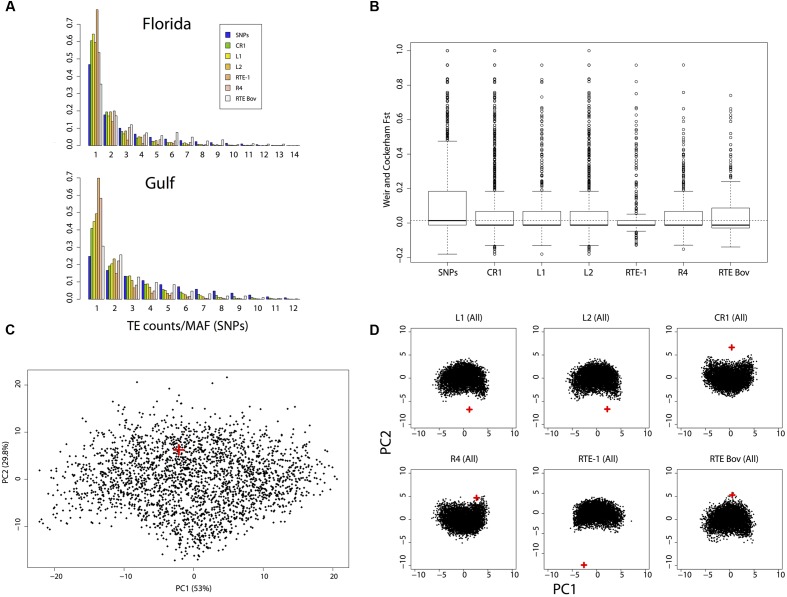
**Summary of allele frequency spectra and simulations.** **(A)** Allele frequency spectra for SNPs and LINE insertions in the East Florida and Gulf-Atlantic populations. For SNPs, the frequency of the minor allele in each population was considered. **(B)** *F*_ST_ distribution for SNPs and transposons clades. The dotted line represents the median for SNPs. **(C)** Principal Component Analysis (PCA) summarizing the joint allele frequency spectrum for SNP simulations. **(D)** PCA obtained after simulating insertion polymorphism in the six main clades. For all PCAs, the red crosses indicate the predicted position of the observed dataset.

Strikingly few insertions occurred at high allelic frequencies or are fixed in either population (**Figure 2A**). We found 16 LINE insertions that were fixed in the East Florida population but absent in the reference genome (12 CR1, three L2 and one R4), and 28 LINE insertions that were fixed in the Gulf population but absent in the reference genome (27 CR1, four L2, two R4 and one L1). Only two insertions were found to be fixed across all the genomes sequenced here but absent in the reference sequence and both were from the CR1 clade. The site frequency spectrum (SFS) of insertions is consistently skewed toward low frequencies when compared to SNPs’ minor allele frequencies (**Figure 2A**), which we used as a proxy for the “neutral” demographic history of the two populations. The only exception to this pattern is RTEBovB, where insertions at intermediate and high frequencies were more common in both populations. The skew in SFS was captured by Tajima’s D, which takes negative values for all categories of LINEs and for both populations, and average pairwise differences over the two populations, which were almost always lower for LINE insertions than for SNPs (**Table 3**). These two statistics are consistent with there being an excess of singletons and rare variants. This pattern was especially strong for RTE-1 and R4 clades in the Gulf population (**Figure 2A**), with a significant reduction in the mean number of pairwise differences even compared with other LINE clades (pairwise comparisons, Wilcoxon rank sum tests, all *P* < 1.7 × 10^-6^). This reduced polymorphism was also reflected by the lower *F*_ST_ values observed for insertions when compared to SNPs (**Figure 2B**). The proportion of alleles found exclusively in Florida (private alleles) was higher than the proportion of private alleles in the Gulf-Atlantic population (Wilcoxon signed rank test on all subgroups in **Table 3**, *V* = 91, *p* = 2.4 × 10^-4^), suggesting a reduced genetic diversity in the Gulf population. Similarly, Tajima’s D was consistently higher in the Gulf population (*V* = 88, *P* = 3.3 × 10^-3^). This pattern was, however, not observed for RTEBovB, which displayed a higher proportion of shared alleles between populations than the other LINEs analyzed here.

### Polymorphic LINE Insertions are Negatively Selected

Estimates of current effective population sizes assessed using the SNP dataset confirmed a large Florida population (diploid population size), and a smaller (but still large) population in Gulf-Atlantic (see **Table 5** for more details). This pattern is consistent with the higher number of polymorphic sites observed in Florida for all markers. Simulated joint SFS based on the demographic model inferred from SNPs were consistent with the observed SFS (**Figure 2C**), suggesting a good fit of the model. Summary statistics obtained from simulations displayed more negative values for Tajima’s D than the ones obtained from random sampling of 100–500 SNPs across the genome. This suggests that our model is conservative for detecting signatures of purifying selection under insertion/drift equilibrium. Nonetheless, observed SFS for LINE insertions never matched the simulations (**Figure 2D**) and the simulated summary statistics such as *F*_ST_ or Tajima’s D were generally larger than the observed ones (**Table 3**). Again, the only exception to this pattern was RTEBovB, which even displayed a higher Tajima’s D than expected in Florida.

**Table 5:** Summary of parameters (in demographic units) estimated with fastsimcoal2.5.

Parameter	2.50%	Maximum Likelihood estimate	97.50%
Ancestral size (Gulf)	379795	1422722	8838592
Ancestral size (Florida)	366002	751115	1756393
Ancestral size (All)	564492	1167977	1488644
Current size (Florida)	1959085	3316203	4603720
Current size (Gulf)	101238	235789	351645
Time since size change (Gulf)	57331	274157	559121
Time since size change (Florida)	275163	802462	1110215
Migration rate (Gulf from Florida)	2.96E-07	3.94E-07	5.51E-07
Migration rate (Florida from Gulf)	2.19E-07	3.38E-07	9.00E-07


*Parameters for modeling insertions were sampled from a uniform distribution bounded by the 95% CI.*

**FIGURE 3:**
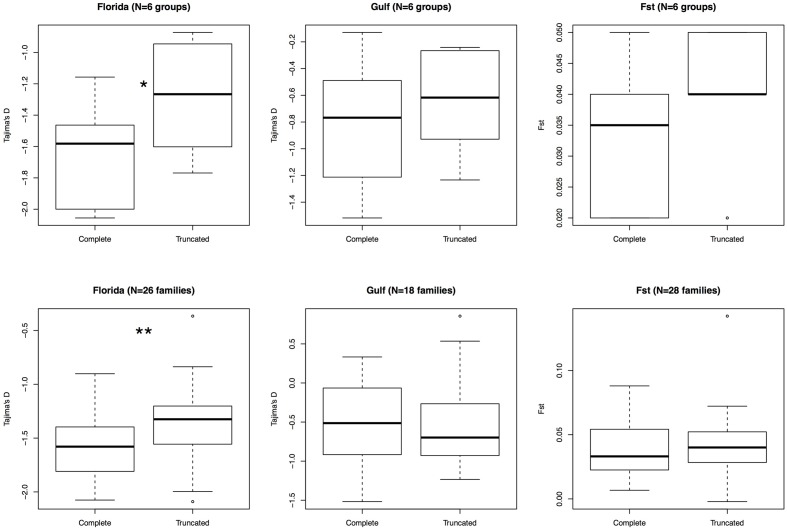
**Comparison of variation between truncated and complete elements.** Six groups were considered: L1 (families AC1 to 16), L1 (families 17 to 20), L2, R4, RTE-1, and CR1 (top row). A more detailed analysis comparing all families within clades is also displayed (bottom). Only polymorphic families with at least 10 polymorphic sites in a population were retained. Wilcoxon signed rank test; ^∗^*P*-value < 0.05, ^∗∗^*P*-value < 0.01.

Since previous studies in other organisms have determined that complete elements are found at lower frequencies than truncated ones, we compared the frequency of these two types of elements. We assessed whether there was any difference between these two categories by comparing Tajima’s D, *F*_ST_ and the mean number of pairwise differences between truncated and complete elements (**Figure 3**). In Florida, Tajima’s D was significantly skewed toward more negative values for complete elements than for truncated ones (26 polymorphic families, *V* = 69, *P*-value = 5.6 × 10^-3^). The average pairwise differences were consistent with this pattern, being always significantly lower for complete elements than for truncated elements in Florida (**Table 6**). In the Gulf-Atlantic population, the values for Tajima’s D tend to be lower for full-length CR1, R4 and RTE1 than for truncated ones, but those differences are not significant. However, the average pairwise differences were significantly different between full-length and truncated elements RTE-1, R4 and CR1 but not for L1 and L2 (**Table 6**).

**Table 6:** Comparison of the mean number of pairwise divergence for complete and truncated elements in the two populations.

Clade	Florida, complete	Florida, truncated	W summary statistics	*P*-value	Gulf, complete	Gulf, truncated	W summary statistics	*P*-value
CR1	**0.209**	**0.225**	**19360000**	**6.41E-07**	**0.297**	**0.313**	**2865600**	**0.001878**
L1 (AC 1 to 16)	**0.213**	**0.249**	**142220**	**6.79E-06**	0.322	0.296	30660	0.06041
L1 (AC 17 to 20)	**0.172**	**0.203**	**57887**	**2.30E-05**	0.276	0.314	8754	0.05928
L2	**0.200**	**0.229**	**2043000**	**5.99E-07**	0.278	0.280	517370	0.8827
R4	**0.234**	**0.254**	**210700**	**0.01054**	**0.246**	**0.266**	**85305**	**0.02438**
RTE01	**0.176**	**0.192**	**403250**	**0.0001458**	**0.225**	**0.245**	**122830**	**0.01861**


*Significance for each comparison between truncated and complete elements was assessed using a Wilcoxon rank sum test. Significant comparisons are highlighted in bold.*

## Discussion

Using whole genome resequencing data, we investigated the population dynamics of polymorphic LINEs in the lizard *A. carolinensis*. We found that LINEs generate a considerable amount of structural polymorphism in this species, in excess of 45,000 insertions, including close to 7,000 full-length elements. This is considerably more than the 998 polymorphic L1 insertions identified by the 1,000 genomes project in the global human population (Stewart et al., 2011) but similar to the number of LINE polymorphisms (∼40,000) found across 17 classical and wild derived mouse strains, which evolution roughly covers a similar time span (∼2 my) (Nellaker et al., 2012). The number of polymorphisms detected here is about four times larger than the number of copies detected in the published genome, which is consistent with the idea that most insertions do not reach fixation (Novick et al., 2009). Additionally, it is important to note that the estimates of LINE polymorphism presented here is likely a conservative one. There are several reasons for this: we used stringent criteria (a maximum of 2% divergence) when identifying LINE insertions, greater depth of coverage could potentially improve the sensitivity of our analyses, and our approach assumes that all insertions in the reference genome are fixed. Together, this will bias our analyses against the identification of rare or degenerate LINE insertions, however, a reduction in this bias would only further support the observations and conclusions described here.

We report substantial differences in the replicative success of LINEs in anoles (**Table 1**). CR1 accounts for more than half of these insertions, followed in abundance by L2, RTE, L1 and R4. Interestingly the total number of insertion generated by a specific clade is not related to the number of potential progenitors. For instance 62% of the ∼2,850 RTE1 insertions, 41% of the ∼1,730 R4 and 43% of the ∼2,500 L1 are complete whereas only 7% of the ∼11,210 L2 and 9% of the ∼27,800 CR1 are complete. This pattern is clearly related to the probability of truncation of LINEs (**Figure 1**). These different patterns of truncation are indicative of variations in the processivity of the reverse-transcription reaction among clades that will need to be further explored experimentally. The inverse relation between copy number and fraction of complete elements suggests that clades are using different strategies to ensure their long-term success. Elements that have a low probability of generating full-length copies (CR1 and L2) tend to generate a much larger number of insertions, increasing the odds that some of these insertions are full-length and potential progenitors. By analogy with the field of ecology, this strategy would be similar to a species with an *r* reproductive strategy, i.e., a strategy where many offspring are produced thus compensating for the low survival to adulthood. In contrast, there is no pressure for L1, RTE1 and R4 to produce a large number of copies since many of the new insertions will be full-length and capable of further transpositions. This is similar to the K strategy where the number of offspring is limited but their chance to propagate the species is high.

In all clades and families examined (with the notable exception of RTEBovB which is discussed below), polymorphic LINE insertions were found at very low frequency and the vast majority were observed from only a single chromosome in our sample. We also showed that the frequency distribution of LINE polymorphisms is significantly skewed toward lower values than the SNP distribution, which presumably reflects the effect of purifying selection acting on LINEs. In addition, we found this skew to be more pronounced for the Floridian population than for the Gulf-Atlantic population and for long elements than for the truncated ones. Purifying selection efficiently prevents the fixation of LINE insertions in anoles because the effective population size of extant and ancestral anole populations is large, ranging from ∼236,000 individuals for the extant Gulf-Atlantic population to ∼3,332,000 for Florida (**Table 5**). Under such demographic conditions, the chance of fixation of a novel insertion, deleterious or neutral, is very low (Gonzalez and Petrov, 2012). In fact, the observation that more private alleles are detected in Florida than in the Gulf population (as well as a higher proportion of polymorphic sites, and a SFS skewed toward low frequencies and singletons) is consistent with Florida’s larger population size compared to the Gulf population (Tollis et al., 2012; Tollis and Boissinot, 2014; Manthey et al., 2016) and is suggestive of a stronger effect of drift on the Gulf-Atlantic population, as previously noted (Tollis and Boissinot, 2013). Thus, the low frequency distribution of LINEs in *A. carolinensis* results both from the effect of selection and a large effective population size. However, previous studies have shown that a number of insertions present in the published genome sequence are fixed (Tollis and Boissinot, 2013). Under the current demographic conditions, it is unlikely that the fixation of the elements occurred recently. Instead it is plausible that these insertions reached fixation when the effective population size of *A. carolinensis* was smaller, possibly at the time of the colonization of North America from Cuba (Glor et al., 2005). Comparison of LINE polymorphisms with genomic sequence from the Cuban species *A. porcatus* and *A. allisoni* will be necessary to answer this question.

The case of RTEBovB is unique among the LINEs analyzed here because it exemplifies the dynamics of a family going extinct. This family is mostly constituted of truncated elements, and is likely ancient (Novick et al., 2009). It displays the highest proportion of shared alleles (49.42%), suggesting that many insertions rose to relatively high frequencies even before the split between populations. It is also the only family for which we observed a higher Tajima’s D than expected, possibly due to ancient demographic variation that is not even captured by the SNPs. The observed pattern is thus consistent with the age of the family and suggests that these elements were not eliminated by selection.

The excess of singletons and the general lower frequency of LINE polymorphisms than SNPs suggest that LINEs are negatively selected and constitute a genetic load for their host. This pattern is consistent with the very low divergence calculated between elements from the same family (Novick et al., 2009; Tollis and Boissinot, 2013) and supports a turnover model in which insertions rarely reach fixation and in which novel insertions are eliminated from the population as new insertions are generated. We also determined that the intensity of selection is stronger against complete elements. This is in line with previous studies in human, fruit fly, and stickleback populations, which showed that selection against TEs is length dependent (Petrov et al., 2003; Boissinot et al., 2006; Blass et al., 2012). However, truncated elements are also found at lower frequency in the populations than expected under neutrality (**Table 3**) suggesting that they are negatively selected. This result contrasts with studies in humans where truncated insertions were shown to behave like neutral alleles (Boissinot et al., 2006). Thus, the negative effect of LINEs does not seem to be limited to long elements in *Anolis*, although those seem to be more deleterious. It was proposed that the deleterious effect of LINEs in vertebrates result mostly from their ability to mediate ectopic recombination leading to chromosomal rearrangements (Furano et al., 2004; Boissinot et al., 2006; Song and Boissinot, 2007; Tollis and Boissinot, 2013), and our observation that complete elements are under stronger purifying selection than truncated ones supports this model. However, the lower frequency of truncated insertions compared with SNPs raises the possibility that ectopic recombination in anoles could also involve short elements, thus providing support to the hypothesis that ectopic recombination may not be as tightly regulated in non-mammalian vertebrates as it is in mammals (Furano et al., 2004; Novick et al., 2009; Tollis and Boissinot, 2013), and that LINEs may impose a stronger genetic load on reptile genomes than they do in mammals.

An alternative explanation for the observed excess of singletons is a departure from transposition-selection equilibrium. Our coalescence simulations implicitly assume a constant mutation/transposition rate. However, it has been shown that transposable elements can go through bursts of transposition, leading to an excess of insertions having the same age. Thus, a recent burst of transposition can also lead to an excess of recent insertions compared to the expectation under equilibrium, even if LINEs are not under purifying selection (Bergman and Bensasson, 2007; Blumenstiel et al., 2014). However, we observed an excess of singletons across all clades (except RTEBovB), which should not be the case unless all families went through a recent, coordinated burst in both populations. In addition, most clades display elements that are shared between the two populations, and were therefore present in the ancestral population, suggesting that the low frequency of these polymorphisms is not caused by a very recent burst. However, differences in the rate of transposition cannot be fully excluded and could contribute to some of the differences we observe. For example, the RTE1 family, which shows the most negative values of Tajima’s D and the most skewed frequency distribution, is also the one with the smallest fraction of shared polymorphism, suggesting that a recent increase in the rate of transposition could contribute to the excess of singletons in this family. From this perspective, the inclusion of other *A. carolinensis* populations should help characterize the extent of shared polymorphism at the species scale, allowing us to better evaluate the likelihood of recent bursts of activity in distinct populations.

Even if non-equilibrium explanations for the excess of rare insertions are considered unlikely (Petrov et al., 2011; Barron et al., 2014), neutral models would benefit from new ways to model the transposition process and provide even more conservative assessments of either negative or positive selection (Bergman and Bensasson, 2007). Future studies should focus in more detail on the relationship between TE frequencies and genomic features such as recombination hotspots, coding and intergenic regions. Combining information about TE position and SNP variation in regions flanking insertion sites is also a powerful way to detect TEs under selection, and should provide fundamental insights into the dynamics of transposable elements in *Anolis* and vertebrates in general.

## Author Contributions

RR and SB designed the project. RR and YB analyzed the data. SB and YB prepared the original artwork. RR, YB, and SB wrote the manuscript. All authors have made substantial intellectual contributions to the research project and approved the final manuscript.

## Conflict of Interest Statement

The authors declare that the research was conducted in the absence of any commercial or financial relationships that could be construed as a potential conflict of interest.

## Footnotes

1 http://nyuad.nyu.edu/en/research/infrastructure-and-support/core-technology-platforms.html

2 http://www.bioinformatics.babraham.ac.uk/projects/fastqc

3 https://www.ncbi.nlm.nih.gov/sra

4 http://broadinstitute.github.io/picard/

5 http://melt.igs.umaryland.edu/

6 https://github.com/vcflib/vcflib

## References

[B1] 1000 Genomes Project Consortium Auton A. Brooks L. D. Durbin R. M. Garrison E. P. Kang H. M. (2015). A global reference for human genetic variation. *Nature* 526 68–74. 10.1038/nature15393 26432245 PMC4750478

[B2] Alfoldi J. Di Palma F. Grabherr M. Williams C. Kong L. Mauceli E. (2011). The genome of the green anole lizard and a comparative analysis with birds and mammals. *Nature* 477 587–591. 10.1038/nature10390 21881562 PMC3184186

[B3] Bao W. Kojima K. K. Kohany O. (2015). Repbase Update, a database of repetitive elements in eukaryotic genomes. *Mob. DNA* 6 11 10.1186/s13100-015-0041-9 PMC4455052 26045719

[B4] Barron M. G. Fiston-Lavier A. S. Petrov D. A. Gonzalez J. (2014). Population genomics of transposable elements in *Drosophila*. *Annu. Rev. Genet.* 48 561–581. 10.1146/annurev-genet-120213-092359 25292358

[B5] Bergman C. M. Bensasson D. (2007). Recent LTR retrotransposon insertion contrasts with waves of non-LTR insertion since speciation in *Drosophila melanogaster*. *Proc. Natl. Acad. Sci. U.S.A.* 104 11340–11345. 10.1073/pnas.0702552104 17592135 PMC2040900

[B6] Blass E. Bell M. Boissinot S. (2012). Accumulation and rapid decay of non-LTR retrotransposons in the genome of the three-spine stickleback. *Genome Biol. Evol.* 4 687–702. 10.1093/gbe/evs044 22534163 PMC3381678

[B7] Blumenstiel J. P. Chen X. He M. Bergman C. M. (2014). An age-of-allele test of neutrality for transposable element insertions. *Genetics* 196 523–538. 10.1534/genetics.113.158147 24336751 PMC3914624

[B8] Boissinot S. Davis J. Entezam A. Petrov D. Furano A. V. (2006). Fitness cost of LINE-1 (L1) activity in humans. *Proc. Natl. Acad. Sci. U.S.A.* 103 9590–9594. 10.1073/pnas.0603334103 16766655 PMC1480451

[B9] Boissinot S. Entezam A. Furano A. V. (2001). Selection against deleterious LINE-1-containing loci in the human lineage. *Mol. Biol. Evol.* 18 926–935. 10.1093/oxfordjournals.molbev.a003893 11371580

[B10] Boissinot S. Sookdeo A. (2016). The evolution of Line-1 in vertebrates. *Genome Biol. Evol.* 8 3485–3507. 10.1093/gbe/evw247 28175298 PMC5381506

[B11] Bolger A. M. Lohse M. Usadel B. (2014). Trimmomatic: a flexible trimmer for Illumina sequence data. *Bioinformatics* 30 2114–2120. 10.1093/bioinformatics/btu170 24695404 PMC4103590

[B12] Campbell-Staton S. C. Goodman R. M. Backstrom N. Edwards S. V. Losos J. B. Kolbe J. J. (2012). Out of Florida: mtDNA reveals patterns of migration and Pleistocene range expansion of the Green Anole lizard (*Anolis carolinensis*). *Ecol. Evol.* 2 2274–2284. 10.1002/ece3.324 23139885 PMC3488677

[B13] Chalopin D. Naville M. Plard F. Galiana D. Volff J. N. (2015). Comparative analysis of transposable elements highlights mobilome diversity and evolution in vertebrates. *Genome Biol. Evol.* 7 567–580. 10.1093/gbe/evv005 25577199 PMC4350176

[B14] Cost G. J. Feng Q. Jacquier A. Boeke J. D. (2002). Human L1 element target-primed reverse transcription in vitro. *EMBO J.* 21 5899–5910. 10.1093/emboj/cdf592 12411507 PMC131089

[B15] Csillery K. Francois O. Blum M. G. B. (2012). abc: an R package for approximate Bayesian computation (ABC). *Methods Ecol. Evol.* 3 475–479. 10.1111/j.2041-210X.2011.00179.x 20488578

[B16] Danecek P. Auton A. Abecasis G. Albers C. A. Banks E. DePristo M. A. (2011). The variant call format and VCFtools. *Bioinformatics* 27 2156–2158. 10.1093/bioinformatics/btr330 21653522 PMC3137218

[B17] DePristo M. A. Banks E. Poplin R. Garimella K. V. Maguire J. R. Hartl C. (2011). A framework for variation discovery and genotyping using next-generation DNA sequencing data. *Nat. Genet.* 43 491–498. 10.1038/ng.806 21478889 PMC3083463

[B18] Dewannieux M. Esnault C. Heidmann T. (2003). LINE-mediated retrotransposition of marked Alu sequences. *Nat. Genet.* 35 41–48. 10.1038/ng1223 12897783

[B19] Dewannieux M. Heidmann T. (2005). L1-mediated retrotransposition of murine B1 and B2 SINEs recapitulated in cultured cells. *J. Mol. Biol.* 349 241–247. 10.1016/j.jmb.2005.03.068 15890192

[B20] Duvernell D. D. Pryor S. R. Adams S. M. (2004). Teleost fish genomes contain a diverse array of L1 retrotransposon lineages that exhibit a low copy number and high rate of turnover. *J. Mol. Evol.* 59 298–308. 10.1007/s00239-004-2625-8 15553085

[B21] Elliott T. A. Gregory T. R. (2015). What’s in a genome? The C-value enigma and the evolution of eukaryotic genome content. *Philos. Trans. R. Soc. Lond. B Biol. Sci.* 370 20140331 10.1098/rstb.2014.0331 PMC4571570 26323762

[B22] Excoffier L. Dupanloup I. Huerta-Sanchez E. Sousa V. C. Foll M. (2013). Robust demographic inference from genomic and SNP data. *PLoS Genet.* 9:e1003905 10.1371/journal.pgen.1003905 PMC3812088 24204310

[B23] Excoffier L. Foll M. (2011). fastsimcoal: a continuous-time coalescent simulator of genomic diversity under arbitrarily complex evolutionary scenarios. *Bioinformatics* 27 1332–1334. 10.1093/bioinformatics/btr124 21398675

[B24] Furano A. V. (2000). The biological properties and evolutionary dynamics of mammalian LINE-1 retrotransposons. *Prog. Nucleic Acid Res. Mol. Biol.* 64 255–294. 10.1016/S0079-6603(00)64007-2 10697412

[B25] Furano A. V. Duvernell D. Boissinot S. (2004). L1 (LINE-1) retrotransposon diversity differs dramatically between mammals and fish. *Trends Genet.* 20 9–14. 10.1016/j.tig.2003.11.006 14698614

[B26] Glor R. E. Losos J. B. Larson A. (2005). Out of Cuba: overwater dispersal and speciation among lizards in the *Anolis carolinensis* subgroup. *Mol. Ecol.* 14 2419–2432. 10.1111/j.1365-294X.2005.02550.x 15969724

[B27] Gonzalez J. Petrov D. A. (2012). Evolution of genome content: population dynamics of transposable elements in flies and humans. *Methods Mol. Biol.* 855 361–383. 10.1007/978-1-61779-582-4_13 22407716

[B28] Kapitonov V. V. Tempel S. Jurka J. (2009). Simple and fast classification of non-LTR retrotransposons based on phylogeny of their RT domain protein sequences. *Gene* 448 207–213. 10.1016/j.gene.2009.07.019 19651192 PMC2829327

[B29] Kim S. Y. Lohmueller K. E. Albrechtsen A. Li Y. Korneliussen T. Tian G. (2011). Estimation of allele frequency and association mapping using next-generation sequencing data. *BMC Bioinformatics* 12:231 10.1186/1471-2105-12-231 PMC3212839 21663684

[B30] Lander E. S. Linton L. M. Birren B. Nusbaum C. Zody M. C. Baldwin J. (2001). Initial sequencing and analysis of the human genome. *Nature* 409 860–921. 10.1038/35057062 11237011

[B31] Langmead B. Salzberg S. L. (2012). Fast gapped-read alignment with Bowtie 2. *Nat. Methods* 9 357–359. 10.1038/nmeth.1923 22388286 PMC3322381

[B32] Li H. Durbin R. (2009). Fast and accurate short read alignment with burrows-wheeler transform. *Bioinformatics* 25 1754–1760. 10.1093/bioinformatics/btp324 19451168 PMC2705234

[B33] Li H. Handsaker B. Wysoker A. Fennell T. Ruan J. Homer N. (2009). The Sequence Alignment/Map format and SAMtools. *Bioinformatics* 25 2078–2079. 10.1093/bioinformatics/btp352 19505943 PMC2723002

[B34] Luan D. D. Korman M. H. Jakubczak J. L. Eickbush T. H. (1993). Reverse transcription of R2Bm RNA is primed by a nick at the chromosomal target site: a mechanism for non-LTR retrotransposition. *Cell* 72 595–605. 10.1016/0092-8674(93)90078-5 7679954

[B35] Malik H. S. Burke W. D. Eickbush T. H. (1999). The age and evolution of non-LTR retrotransposable elements. *Mol. Biol. Evol.* 16 793–805. 10.1093/oxfordjournals.molbev.a026164 10368957

[B36] Manthey J. D. Tollis M. Lemmon A. R. Moriarty Lemmon E. Boissinot S. (2016). Diversification in wild populations of the model organism *Anolis carolinensis*: a genome-wide phylogeographic investigation. *Ecol. Evol.* 6 8115–8125. 10.1002/ece3.2547 27891220 PMC5108263

[B37] Martin S. L. Li W.-H. P. Furano A. V. Boissinot S. (2005). The structures of mouse and human L1 elements reflect their insertion mechanism. *Cytogenet. Genome. Res.* 110 223–228. 10.1159/000084956 16093676

[B38] McClure M. A. Richardson H. S. Clinton R. A. Hepp C. M. Crowther B. A. Donaldson E. F. (2005). Automated characterization of potentially active retroid agents in the human genome. *Genomics* 85 512–523. 10.1016/j.ygeno.2004.12.006 15780754

[B39] Myers S. Bottolo L. Freeman C. McVean G. Donnelly P. (2005). A fine-scale map of recombination rates and hotspots across the human genome. *Science* 310 321–324. 10.1126/science.1117196 16224025

[B40] Neafsey D. E. Blumenstiel J. P. Hartl D. L. (2004). Different regulatory mechanisms underlie similar transposable element profiles in pufferfish and fruitflies. *Mol. Biol. Evol.* 21 2310–2318. 10.1093/molbev/msh243 15342795

[B41] Nellaker C. Keane T. M. Yalcin B. Wong K. Agam A. Belgard T. G. (2012). The genomic landscape shaped by selection on transposable elements across 18 mouse strains. *Genome Biol.* 13:R45 10.1186/gb-2012-13-6-r45 PMC3446317 22703977

[B42] Novick P. A. Basta H. Floumanhaft M. McClure M. A. Boissinot S. (2009). The evolutionary dynamics of autonomous non-LTR retrotransposons in the lizard *Anolis carolinensis* shows more similarity to fish than mammals. *Mol. Biol. Evol.* 26 1811–1822. 10.1093/molbev/msp090 19420048

[B43] Ohshima K. Hamada M. Terai Y. Okada N. (1996). The 3’ ends of tRNA-derived short interspersed repetitive elements are derived from the 3’ ends of long interspersed repetitive elements. *Mol. Cell. Biol.* 16 3756–3764. 10.1128/MCB.16.7.3756 8668192 PMC231371

[B44] Ostertag E. M. Kazazian H. H. Jr. (2001). Twin priming: a proposed mechanism for the creation of inversions in L1 retrotransposition. *Genome Res.* 11 2059–2065. 10.1101/gr.205701 11731496 PMC311219

[B45] Petrov D. Aminetzach Y. T. Davis J. C. Bensasson D. Hirsh A. E. (2003). Size matters: non-LTR retrotransposable elements and ectopic recombination in *Drosophila*. *Mol. Biol. Evol.* 20 880–892. 10.1093/molbev/msg102 12716993

[B46] Petrov D. A. Fiston-Lavier A. S. Lipatov M. Lenkov K. Gonzalez J. (2011). Population genomics of transposable elements in *Drosophila melanogaster*. *Mol. Biol. Evol.* 28 1633–1644. 10.1093/molbev/msq337 21172826 PMC3080135

[B47] Pfeifer B. Wittelsburger U. Ramos-Onsins S. E. Lercher M. J. (2014). PopGenome: an efficient Swiss army knife for population genomic analyses in R. *Mol. Biol. Evol.* 31 1929–1936. 10.1093/molbev/msu136 24739305 PMC4069620

[B48] Piskurek O. Nishihara H. Okada N. (2009). The evolution of two partner LINE/SINE families and a full-length chromodomain-containing Ty3/Gypsy LTR element in the first reptilian genome of *Anolis carolinensis*. *Gene* 441 111–118. 10.1016/j.gene.2008.11.030 19118606

[B49] Rishishwar L. Marino-Ramirez L. Jordan I. K. (2016). Benchmarking computational tools for polymorphic transposable element detection. *Brief. Bioinform.* 10.1093/bib/bbw072 [Epub ahead of print]. PMC5808724 27524380

[B50] Shen J. J. Dushoff J. Bewick A. J. Chain F. J. Evans B. J. (2013). Genomic dynamics of transposable elements in the western clawed frog (Silurana tropicalis). *Genome Biol. Evol.* 5 998–1009. 10.1093/gbe/evt065 23645600 PMC3673623

[B51] Song M. Boissinot S. (2007). Selection against LINE-1 retrotransposons results principally from their ability to mediate ectopic recombination. *Gene* 390 206–213. 10.1016/j.gene.2006.09.033 17134851

[B52] Stewart C. Kural D. Stromberg M. P. Walker J. A. Konkel M. K. Stutz A. M. (2011). A comprehensive map of mobile element insertion polymorphisms in humans. *PLoS Genet.* 7:e1002236 10.1371/journal.pgen.1002236 PMC3158055 21876680

[B53] Sudmant P. H. Rausch T. Gardner E. J. Handsaker R. E. Abyzov A. Huddleston J. (2015). An integrated map of structural variation in 2,504 human genomes. *Nature* 526 75–81. 10.1038/nature15394 26432246 PMC4617611

[B54] Tajima F. (1989). Statistical method for testing the neutral mutation hypothesis by DNA polymorphism. *Genetics* 123 585–595. 2513255 10.1093/genetics/123.3.585 PMC1203831

[B55] Tollis M. Ausubel G. Ghimire D. Boissinot S. (2012). Multi-locus phylogeographic and population genetic analysis of *Anolis carolinensis*: historical demography of a genomic model species. *PLoS ONE* 7:e38474 10.1371/journal.pone.0038474 PMC3369884 22685573

[B56] Tollis M. Boissinot S. (2012). The evolutionary dynamics of transposable elements in eukaryote genomes. *Genome Dyn* 7 68–91. 10.1159/000337126 22759814

[B57] Tollis M. Boissinot S. (2013). Lizards and LINEs: selection and demography affect the fate of L1 retrotransposons in the genome of the green anole (*Anolis carolinensis*). *Genome Biol. Evol.* 5 1754–1768. 10.1093/gbe/evt133 24013105 PMC3787681

[B58] Tollis M. Boissinot S. (2014). Genetic variation in the green anole lizard (*Anolis carolinensis*) reveals island refugia and a fragmented Florida during the quaternary. *Genetica* 142 59–72. 10.1007/s10709-013-9754-1 24379168 PMC4778398

[B59] Van der Auwera G. A. Carneiro M. O. Hartl C. Poplin R. Del Angel G. Levy-Moonshine A. (2013). From FastQ data to high confidence variant calls: the genome analysis toolkit best practices pipeline. *Curr. Protoc. Bioinformatics* 43 11–33. 10.1002/0471250953.bi1110s43 25431634 PMC4243306

[B60] Volff J. N. Bouneau L. Ozouf-Costaz C. Fischer C. (2003). Diversity of retrotransposable elements in compact pufferfish genomes. *Trends Genet.* 19 674–678. 10.1016/j.tig.2003.10.006 14642744

[B61] Waterston R. H. Lindblad-Toh K. Birney E. Rogers J. Abril J. F. Agarwal P. (2002). Initial sequencing and comparative analysis of the mouse genome. *Nature* 420 520–562. 10.1038/nature01262 12466850

[B62] Wei W. Gilbert N. Ooi S. L. Lawler J. F. Ostertag E. M. Kazazian H. H. (2001). Human L1 retrotransposition: cis preference versus trans complementation. *Mol. Cell. Biol.* 21 1429–1439. 10.1128/MCB.21.4.1429-1439.2001 11158327 PMC99594

